# A comparison of cognitive-behavioral couple therapy and lidocaine in the treatment of provoked vestibulodynia: study protocol for a randomized clinical trial

**DOI:** 10.1186/1745-6215-15-506

**Published:** 2014-12-23

**Authors:** Serena Corsini-Munt, Sophie Bergeron, Natalie O Rosen, Marc Steben, Marie-Hélène Mayrand, Isabelle Delisle, Pierre McDuff, Leen Aerts, Marie Santerre-Baillargeon

**Affiliations:** Department of Psychology, Université de Montréal, 2900 Boulevard Édouard-Montpetit, Montréal, H3T 1J4 Canada; Department of Psychology and Neuroscience, Dalhousie University, Life Sciences Centre, 1355 Oxford Street, Halifax, B3H 4R2 Canada; Department of Obstetrics and Gynecology, IWK Health Centre, 5980 University Avenue, Halifax, Nova Scotia B3K 6R8 Canada; Clinique A, rue McGill, 407 rue McGill, Montréal, QC H2Y 2G3 Canada; Departments of Obstetrics and Gynecology and Social and Préventive Medicine, Université de Montréal and CRCHUM, 850, rue St-Denis, Bureau S02-438, Montreal, Québec H2X 0A9 Canada

**Keywords:** Randomized clinical trial, Vulvodynia, Cognitive-behavioral therapy, Topical lidocaine, Chronic pain, Women’s sexual health, Sexual function, Couple therapy, Treatment outcome, Dyspareunia

## Abstract

**Background:**

Provoked vestibulodynia (PVD), a frequent form of chronic genital pain, is associated with decreased sexual function for afflicted women, as well as impoverished sexual satisfaction for women and their partners. Pain and sexuality outcomes for couples with PVD are influenced by interpersonal factors, such as pain catastrophizing, partner responses to pain, ambivalence over emotional expression, attachment style and perceived relationship and sexual intimacy. Despite recommendations in the literature to include the partner in cognitive-behavioral therapy targeted at improving pain and sexuality outcomes, no randomized clinical trial has tested the efficacy of this type of intervention and compared it to a first-line medical intervention.

**Methods:**

This bi-center, randomized clinical trial is designed to examine the efficacy of cognitive-behavioral couple therapy compared to topical lidocaine. It is conducted across two Canadian university-hospital centers. Eligible women diagnosed with PVD and their partners are randomized to one of the two interventions. Evaluations are conducted using structured interviews and validated self-report measures at three time points: Pre-treatment (T1: prior to randomization), post-treatment (T2), and 6-month follow-up (T3). The primary outcome is the change in reported pain during intercourse between T1 and T2. Secondary outcomes focus on whether there are significant differences between the two treatments at T2 and T3 on (a) the multidimensional aspects of women’s pain and (b) women and partners’ sexuality (sexual function and satisfaction), psychological adjustment (anxiety, depression, catastrophizing, self-efficacy, and quality of life), relationship factors (partner responses and dyadic adjustment) and self-reported improvement and treatment satisfaction. In order to detect an effect size as small as 0.32 for secondary outcomes, a sample of 170 couples is being recruited (27% dropout expected). A clinically significant decrease in pain is defined as a 30% reduction.

**Discussion:**

The randomized clinical trial design is the most appropriate to examine the efficacy of cognitive-behavioral couple therapy, a recently developed and pilot-tested psychosocial intervention for couples coping with PVD, in comparison to a frequent first-line treatment option, topical lidocaine. Findings from this study will provide important information about empirically supported treatment options for PVD, and inform future treatment development and research for this patient population.

**Trial registration:**

Clinicaltrials.gov NCT01935063; registration date: 27 August 27 2013.

## Background

Chronic pain problems involving the female reproductive system represent major health concerns for women. Often misunderstood and misdiagnosed or ignored, gynecologic or genital pain conditions carry a heavy personal cost to patients and a significant financial burden to society, with women consulting as many as 4 to 6 physicians about the pain [1]. Vulvodynia, or chronic unexplained vulvar pain, is an example of female genital pain. Recent population-based surveys suggest that, by 40 years of age, 7 to 8% of women report vulvodynia-like symptoms [2]. Provoked vestibulodynia (PVD) – an acute recurrent pain localized within the vulvar vestibule and experienced primarily during intercourse – is suspected to be the most frequent type of vulvodynia in premenopausal women [3].

While not one clear etiologic pathway exists for all women with PVD, continuing research points to a multifactorial understanding, with certain factors presenting as more common among women with PVD compared to women without this type of pain. These factors include early menarche (younger than or equal to 11 years of age [4]), repeated yeast infections [5], polymorphisms in genes regulating inflammatory response [6], nociceptor proliferation and sensitization [7, 8], lower touch and pain thresholds [9], and pelvic floor muscle dysfunction [10]. The essential result is that the pain modulation process is less efficient in women with PVD [11]. Extending beyond the biological to accommodate a biopsychosocial model of pain, there is a growing body of research highlighting the significance of psychosocial factors as robust predictors of pain and associated disability.

Cross-sectional research with a sample of women with PVD showed that increased hypervigilance to and fear of pain, and higher pain catastrophizing were significantly associated with increased pain experiencing during intercourse [12]. From this same study, greater anxiety and avoidance were associated with poorer sexual function, and lower pain self-efficacy was related to worse pain and sexual function outcomes. As for interpersonal factors, partner responses to pain are thought to reinforce and perpetuate the pain experience of patients [13–16]. The most studied types of partner responses to PVD are solicitous (demonstrations of sympathy), negative (demonstrations of anger), and facilitative (encouraging adaptive coping). In a cross-sectional examination, increased solicitous and decreased facilitative partner responses were associated with higher pain intensity, and lower negative and higher facilitative partner responses were associated with increased sexual satisfaction for women [13, 17]. Further examination of these relationships indicated that the associations were respectively mediated by pain catastrophizing and relationship satisfaction [14]. Using a daily-diary design, it was found that women reported improved sexual functioning on days when they perceived partner responses to pain as more facilitative [16]. Therefore, cognitions and behaviors relating to pain, such as pain self-efficacy, catastrophizing and partner responses to pain, represent avenues through which interventions might target pain and sexuality outcomes.

More affective interpersonal factors have also shown to be related to pain and sexual outcomes. Among a sample of couples with PVD, women’s self-reported relationship and sexual intimacy were significantly associated with their sexual function, sexual satisfaction and pain self-efficacy, suggesting the potential protective influence of a couple’s intimacy for sexual well-being in the context of pain [18]. Similarly, low ambivalence over emotional expression in both partners, indicating they are comfortable with the way they express emotions, was significantly linked to better psychological, sexual and relational outcomes [19]. Further examination of dyadic factors related to PVD has shown that the association between a woman’s insecure attachment style and lower sexual functioning was mediated by lower levels of sexual assertiveness [20]. Taken together, these studies highlight the importance of fostering communication, both expression and assertion, in couples coping with PVD. Although empirical evidence continues to mount in support of the important role of relational variables in the pain and psychosexual sequelae of PVD, many treatment options target the pain primarily and no study to date has examined the efficacy of a treatment that incorporates systematic inclusion of the partner.

Despite the wide variety of treatment options, there is a dearth of prospective, controlled studies assessing their efficacy. Localized interventions include topical lidocaine [21], biofeedback [22], pelvic floor physical therapy [23], topical use of estradiol and testosterone compound [24] and vestibulectomy (surgery) [25]. Systemic interventions include tricyclic antidepressants [26]. Psychotherapeutic interventions include cognitive behavioral therapy (CBT) focusing on reducing pain and improving sexuality [27]. Topical lidocaine is currently recommended as an effective first-line intervention for PVD [28–30]. Two surveys confirmed that a local anesthetic, and/or local measures including lidocaine, are the most commonly used intervention (89% and 83.8%, respectively), with 52% of physicians choosing lidocaine as a first-line therapy [31, 32]. Lidocaine is thought to act peripherally by reducing nociceptor sensitization [33]. Zolnoun and colleagues [34] hypothesized that long-term use of overnight topical lidocaine may minimize feedback amplification of pain, and their prospective study found that nightly applications of 5% lidocaine resulted in a significant pre- to post-treatment decrease in self-reported pain and an increase in intercourse frequency. A randomized trial comparing topical lidocaine and electromyographic biofeedback showed that both treatments yielded significant decreases in vestibular pain pressure thresholds and improved sexual functioning [22]. Using the tampon-test (that is, change in pain experience during the insertion and removal of a tampon, on a scale of 0 to 10), a randomized, double-blinded, placebo controlled trial examining the differential efficacy of lidocaine and the tricyclic antidepressant desipramine, showed that none of the active treatment arms demonstrated significantly greater pain reductions than the placebo, with all treatment arms resulting in pre- to post-treatment pain decreases [33]. However, the study design had limitations including a sample size smaller than statistically recommended, perhaps obscuring treatment effect. Further, 21 to 38% of women reported no sexual activity during the 12-week trial [33], suggesting that this sample may not be representative of partnered women who remain sexually active. Moreover, the tampon-test is not representative of the pain a woman experiences during intercourse. Given the multifaceted nature of the etiology and impact of PVD, a treatment that can target pain and also psychological, sexual, and relationship consequences would have a presumed advantage over one targeting only biomedical aspects of PVD.

In the first randomized clinical trial (RCT) examining treatment for PVD, vestibulectomy demonstrated the most significant reductions in pain during intercourse at post-treatment; however, all interventions, including group-CBT, showed positive outcomes for sexual function and psychological adjustment [27]. At 2.5 year follow-up, women assigned to CBT did not differ from those assigned to vestibulectomy in terms of pain during intercourse [35]. Another RCT examining the efficacy of individual CBT for vulvar pain compared to a supportive psychotherapy demonstrated that CBT yielded significantly greater improvements in pain and sexual function from pre- to post-treatment, with gains maintained at 1 year follow-up [36]. These findings suggest that CBT may yield a positive impact on more dimensions of PVD than does a first-line medical treatment.

A systematic review of PVD treatment studies concluded that because behavioral treatments yield comparable success to several medical interventions but with no negative side effects, CBT represents an encouraging non-invasive option that can target pain as well as psychosexual consequences experienced by the woman and her partner [25]. However, while CBT for PVD has been successfully investigated in group and individual format, it has never been examined in couple format. Moreover, CBT formatted for the couple is the most common and recommended way that CBT for sexual dysfunction is delivered in clinical settings, hence the form of CBT that is most representative of clinical reality [37].

The growing body of work focusing on the interpersonal aspects of PVD has led to the development of a novel, targeted cognitive-behavioral couple therapy (CBCT). CBCT was pilot-tested for feasibility and preliminary effectiveness, showing significant pre- to post-treatment improvements in pain during intercourse and sexual function for women with PVD, and sexual satisfaction for both members of the couple [38]. Therefore, the primary goal of the present RCT is to evaluate the efficacy of CBCT in comparison to one of the most commonly prescribed first-line medical interventions, topical lidocaine, in the reduction of pain during intercourse at post-treatment. Secondary research goals include the examination of differences between the two treatments at post-treatment and 6 month follow-up on: (a) the multidimensional aspects of women’s pain; (b) women and partners’ sexuality (sexual function and satisfaction, frequency of intercourse); (c) psychological adjustment (anxiety, depression, catastrophizing, self-efficacy, attributions and quality of life); (d) relationship factors (partner responses and dyadic adjustment); and (e) self-reported improvement and treatment satisfaction. Childhood trauma and co-morbid pain conditions are being considered as moderators of treatment response.

## Methods/design

### Design

The present study is a bi-center trial using an intent-to-treat analysis strategy, designed to compare the efficacy of CBCT and topical lidocaine for the treatment of PVD. This design is based on previously conducted RCTs assessing treatments for PVD and recommendations outlined in the Initiative on Methods, Measurement, and Pain Assessment in Clinical Trials (IMMPACT) guidelines for chronic pain clinical trials [39]. This trial is comprised of three evaluation points (pre-treatment, post-treatment and 6 month follow-up) carried out via structured interviews and online validated self-report questionnaires.

### Research sites

This research is supported by an operating grant from the Canadian Institutes of Health Research, and has ethical approval from the Research Ethics Committee of the Centre Hospitalier de l’Université de Montréal (13.156) and the IWK Health Centre Research Ethics Board (1014930). The study involves collaborations from researchers from the following institutions: Centre Hospitalier de l’Université de Montréal, the Université de Montréal, the IWK Health Centre, and Dalhousie University.

### Participants

Participant eligibility criteria are described in Table 1. These criteria ensure recruitment of a homogeneous sample of sexually active women diagnosed with PVD. As part of the eligibility assessment, a comprehensive gynecologic examination is conducted based on a standardized protocol. This protocol, successfully used in a previous RCT [27], is outlined in Table 2. Women with PVD who present with a concomitant infection are treated and then asked to come in again to repeat the gynecologic protocol to determine eligibility.

**Table 1:** **Eligibility criteria**

Level 1	Level 2	Level 3
Inclusion criteria	Inclusion criteria	Exclusion criteria
Participants with PVD	Participants with PVD	Participants with PVD
- Pain during intercourse which a) is subjectively distressing, b) occurs on 80% of intercourse attempts, and c) has lasted for at least 1 year	- Significant pain in one or more locations of the vestibule during the gynecological examination, which is operationalized as a minimum patient pain rating of 4 on a scale of 0 to 10	- Vulvar pain not clearly linked to intercourse or pressure applied to the vestibule
- Pain limited to intercourse and other activities involving pressure to the vestibule	- Diagnosis of PVD	- Presence of one of the following: a) active infection, b) vaginismus (as defined by DSM-IV), e) dermatologic lesion, f) pregnancy or planning a pregnancy, g) known allergy to lidocaine, and h) menopause.
- Sexually active as a couple in the last 3 months (not limited to but must include some attempted vaginal penetration)		- Receiving treatment for PVD
- Cohabiting and/or been a couple for at least 6 months and have at least 4 in-person contacts per week		**Participants with PVD and Partners**
- Aged 18–45 years		- Presence of major medical and/or psychiatric illness in either partner
**Participants with PVD and Partners**		- Receiving couple therapy
- Read and write in English and/or French, with regular access to internet and email		- Presence of severe relational distress and/or high level of physical conflict
- Age: 18 years or older		

*DSM-IV* Diagnostic and Statistical Manual of Mental Disorders-IV; *PVD* provoked vestibulodynia.

**Table 2:** **Gynecology examination protocol**

- Brief interview about past medical history, medication, and obstetrical/gynecological history, including painful intercourse	
- A one digit single-handed palpation of the following areas: vagina, uterus and adnexa	
- A standard bimanual palpation of the uterus and adnexa	
- Physician to record participants’ pain rating at each site on a scale of 0 (no pain) to 10 (worst pain ever)	
- Physician to note any other physical findings, and note diagnosis	

### Treatments

#### Cognitive-behavioral couple therapy

CBCT is delivered over 12 weeks, with couples attending a 75-minute therapy session once per week. CBCT was adapted from Bergeron and colleagues Cognitive-Behavioral Pain and Sex Therapy manual [40] – a treatment manual outlining a CBT group-therapy for women with PVD – to include recent and pertinent findings about pain-related, sexuality, interpersonal and psychological factors associated with PVD. Throughout the study, CBCT therapists take part in weekly supervision sessions with a psychologist with training and expertise in CBCT for pain and sexuality. Adherence to the treatment manual is monitored via DVD recordings of therapy sessions, which are reviewed on an ongoing basis by the principal investigators. If any deviations are noted, therapists are given additional supervision. An outline of select CBCT interventions is provided in Table 3. No adverse reactions associated with CBT for PVD have been noted in the literature, yet all participants are instructed to contact research personnel should they experience any adverse events as part of treatment.

**Table 3:** **Cognitive-behavioral couple therapy treatment outline**

Session	In-session interventions	Homework exercises
1	Discuss treatment expectations	Pain and sex journaling
2	Psychoeducation re: provoked vestibulodynia	Mindfulness breathing
3	Communication: disclosure and validation	
4	Identifying biopsychosocial factors influencing pain	Pain-localization
5	Role of anxiety for pain and sex	Kegel exercises (if appropriate)
6	Partner and woman responses to pain	Sensate focus
7	Redefining the sexual narrative	Dilatation exercises (if applicable)
8	Facilitating sexual desire and arousal	
9	Psychoeducation re: pain attributions	
10	Cognitive defusion and meditation	Cognitive defusion
11	Importance of self-assertion	Homework choice
12	Discussion: information learned and tools for the future	

#### Topical lidocaine

Participants perform nightly applications of a 5% lidocaine ointment on the vulvar vestibule, at the entry of the vagina (50 mg/g, Lidocaine ointment 5% USP Lidodan, Odan, tubes of 35 g) for 12 weeks, as described by Zolnoun and colleagues [34]. In addition, the ointment is applied to a cotton square kept on the vestibule via the participant’s underwear overnight to ensure continued 7- to 8-hour contact. Appropriate written and oral instructions are provided to participants (Table 4). A research assistant conducts standardized weekly phone calls in order to monitor potential adverse events. Participants are instructed to inform research personnel if they experience any adverse reactions. Potential side effects of lidocaine include: skin irritation such as redness, itching, swelling, burning sensation, and prickling sensation.

**Table 4:** **Lidocaine application instructions**

***At bedtime***	
Step 1	First wash hands thoroughly then make sure that targeted region is also clean. Dry by dabbing region with a towel (avoid vigorously rubbing)
Step 2	Apply a small quantity of ointment (the size of a marble) directly on the vulvar vestibule (see the diagram on the next page). Next, fold the cotton gauze in 4 (to make a smaller square) and apply the same amount of ointment on the gauze (size of a marble)
	You may want to use a mirror to help guide you
Step 3	Cotton underwear may help keep the cotton gauze in place overnight while you sleep. You want to try to keep the lidocaine ointment in contact with the painful part of your vulvar vestibule for about 8 hours. Remove it when you wake up
Step 4	Wash hands immediately to avoid spreading ointment on unwanted areas
Step 5	Repeat these steps everyday for 12 weeks and fill out your *Daily Lidocaine Log* everyday!
Note: If you have to use the washroom during the night, repeat these steps to ensure that the ointment is present for the rest of the night.	

#### Monitoring during treatment

Partner participation is an integral part of the study, independent of treatment arm. For CBCT, overall participant-treatment adherence is measured by monitoring attendance to CBCT sessions and by asking participants at each session to rate the frequency of weekly practice of homework exercises. Participants who attend less than 75% of sessions and do less than 50% of the homework exercises are considered non-compliant. These numbers are based on the fact that therapy sessions are an essential component of treatment whereas it is not clear up to what point homework contributes to outcome [27]. Therapy attendance and homework completion are standard aspects of couple therapy, addressed actively by the therapist at each session, namely by providing a rationale for each homework exercise and problem-solving before and after taking part in the exercise. Missed therapy sessions are rescheduled if notice is provided and at the convenience of the participants. In order to empirically document therapist adherence to the treatment manual (treatment reliability), two trained research assistants are independently viewing and coding a random sample of videotapes representing a quarter of all therapy sessions. For the topical lidocaine condition, the weekly phone calls to participants to monitor adverse events are also intended to facilitate compliance with the application and minimize the risk of drop-outs. Participants assigned to this condition also complete a daily diary to document the lidocaine application to determine treatment reliability. Participants who apply the lidocaine less than 75% of the total evenings comprising the treatment period are considered non-compliant.

### Recruitment and follow-up

Participants are being recruited via four centers, all specialized in the assessment and treatment of vulvo-vaginal pain: 1) Centre hospitalier de l’Université de Montréal, pavillon St-Luc, Clinique Vuva, directed by MHM, 2) Clinique A rue McGill, a sexual health clinic directed by MS, 3) the IWK Health Centre in Halifax where ID holds a general gynecology practice, and 4) the Queen Elizabeth II Health Sciences Centre in Halifax where ID directs a specialized gynecology-dermatology vulvar clinic. Additionally, women diagnosed with PVD during clinic visits with collaborating physicians are informed of the study and given the choice to participate after they have been fully informed of other available treatments. Additionally, announcements are being placed online and in Montreal and Halifax newspapers describing the study, and flyers and pamphlets are placed in other gynecology offices of both cities and posted on university and college bulletin boards. Women agreeing to participate are asked to confirm their partners’ participation and are scheduled for a pre-treatment evaluation, including a gynecological examination. Both partners sign the consent form. Following satisfaction of inclusion and exclusion criteria, eligible couples are randomized to one of the two treatment options.

### Randomization and blinding

Randomization takes place shortly before treatment initiation (maximum 2-week delay). Participants are screened across three levels (see Table 1) for eligibility criteria and data are entered in the electronic eligibility check web form created using Dacima™ Clinical Suite (Dacima Software Inc., Montreal, Quebec, Canada). Participants meeting eligibility criteria are randomized to either CBCT or lidocaine, such that an approximately equal number of CBCT and lidocaine participants are obtained (that is, individual level randomization is used with stratification by site). The randomization sequence was written to generate random permuted blocks with block sizes of four, six and eight to make the sequence difficult to predict without leading to a major imbalance in numbers between treatment groups if a block is incomplete at the end of recruitment. Participants found ineligible are excluded and marked as such. To keep interviewers and assessors blind to the treatment condition, participants are instructed not to reveal the treatment to which they were assigned at multiple time points: in the consent form, the information pamphlet provided at randomization and at the time of each assessment.

### Outcomes and moderators

Measures were selected based on the need to assess the multiple dimensions of PVD and the potential impact of treatments on these different dimensions.

#### Pre-treatment moderators

Trauma, a potential moderator of treatment response, is assessed using the Childhood Trauma Questionnaire, a 28-item self-report measure focusing on emotional, physical, and sexual abuse, as well as physical and emotional neglect in childhood [41]. Scores range from 5 to 25 for each type of abuse and a total severity scale can also be computed. Co-morbid pain conditions, another potential moderator for participants with PVD, are assessed during the structured interview.

#### Primary outcome measure

Pain during intercourse is assessed using a visual analog scale ranging from 0 to 10, where 0 is no pain at all, and 10 is the worst pain ever, as recommended by the IMMPACT guidelines for chronic pain clinical trials [39]. Participants report on the average pain experienced in the preceding month. This measure has been shown to detect significant treatment effects in women with PVD [27] and demonstrates a significant positive correlation with other pain intensity measures. Pain during intercourse is the main symptom of PVD and the one that most interferes with quality of life, hence the most relevant measure of functional outcome.

#### Secondary outcome measures for participants with provoked vestibulodynia only

Sociodemographic and vulvo-vaginal pain characteristics are assessed using a structured interview designed specifically for these purposes and successfully used in previous research [27]. During this interview, self-reported monthly frequency of intercourse and co-morbid pain conditions are also assessed.

Pain is also assessed using the McGill Pain Questionnaire (MPQ) [42], which measures the sensory, affective and evaluative components of pain. The MPQ, a widely used adjective checklist, assesses both qualitative and quantitative aspects of pain. The Pain Rating Index of the MPQ scale is also being used.

#### Secondary outcome measures for participants with provoked vestibulodynia and their partners

Partners are also asked to report on their own experiences, such as anxiety and depression symptoms. When measures relate to pain, such as pain catastrophizing and pain attributions, they are reporting on their own catastrophizing and attributions about the woman’s pain. The only exception is self-efficacy, where the partner rates his or her perception of the woman’s self-efficacy vis-à-vis her pain symptoms.

Partner responses to pain from the perspectives of the women with PVD and their partners are measured with the West Haven-Yale Multidimensional Pain Inventory – Significant Other Response Scale (MPI) [43], and the Spouse Response Inventory – Facilitative subscale (SRI) [44], which have been adapted to our PVD population and their partners. These include negative responses, solicitous responses, and distracting responses for the MPI and facilitative responses for the SRI. Internal consistency analyses show alphas ranging from 0.75 to 0.82 for each subscale of partner and patient versions of the MPI and of 0.87 for patient and partner versions of the SRI [14]. Factor analyses have confirmed that the structures of the adaptations to couples facing PVD are the same as that of the original questionnaires. The reliability and validity of both questionnaires have been widely reported [43–45]. A partner version of these scales has recently been validated [46] and used successfully in recent studies [13, 14]. Each scale is analyzed separately.

Dyadic adjustment is assessed using the Couple Satisfaction Index, a 32-item measure of relationship satisfaction, which demonstrates strong convergent validity, and high precision and power for detecting distinctions in levels of satisfaction [47]. Unlike similar relationship satisfaction scales, the Couple Satisfaction Index has been tested with a sample of couples in varying relationship states (for example, dating, engaged, married).

Pain catastrophizing is assessed using the Pain Catastrophizing Scale [48], which consists of 13 items scored on a 5-point scale with the end points (0) ‘not at all’ and (4) ‘all the time’. The Pain Catastrophizing Scale is divided into three subscales: rumination, magnification and helplessness. It is a reliable and valid measure that has demonstrated a stable factorial structure across clinical and general populations [49], and the validated partner version also shows excellent psychometric properties [50].

Pain attributions are measured with the Extended Attributional Style Questionnaire (EASQ) [51], adapted for use with women who experience genital pain and their partners. The adapted EASQ consists of 12 hypothetical negative situations that occur within a genital pain context, and participants are asked to indicate the major cause of the situation (open-ended), and then rate the cause on the following dimensions: internal, global, and stable on a 7-point Likert scale. The EASQ adapted for genital pain demonstrates good internal consistency (alpha = 0.84 to 0.86) for subscales and total score, as well as a similar factor structure to the original EASQ [52].

Anxiety is assessed using the Trait Anxiety scale (20-items) of the Spielberger State-Trait Anxiety Inventory [53]. The State-Trait Anxiety Inventory is a 40-item, well-known and widely used measure of state and trait anxiety that has demonstrated very good psychometric properties across populations [54].

Depression is measured via the Beck Depression Inventory-II, comprised of 21 items, with scores for most items ranging from 0 (low intensity) to 3 (high intensity) [55]. The Beck Depression Inventory-II has been validated for use in chronic pain populations [56].

Pain self-efficacy is assessed using the Painful Intercourse Self-Efficacy Scale [12], which was adapted from the Arthritis Self-Efficacy Scale [57]. The Painful Intercourse Self-Efficacy Scale consists of 20 items with three subscales: self-efficacy for controlling pain during intercourse, for sexual function, and for other symptoms. Participants indicate their perceived ability to carry out sexual activity or to achieve outcomes in pain management by responding on a scale from 10 (very uncertain) to 100 (very certain). Higher scores indicate greater self-efficacy. The reliability and validity of the original version have been established [57] and the factor structure of the adapted version has been shown to be identical to that of the original [12]. Partners complete an adapted version with reference to their beliefs about the woman’s self-efficacy in the same situations.

Quality of life is measured using the Quality Metric™ Short Form 12-question Health Survey. This is a shortened version of the widely-used Short Form-36 health survey and assesses physical and mental health and wellness [58]. The IMMPACT guidelines for pain clinical trials recommend the assessment of quality of life [39].

Sexual function is assessed using the Derogatis Interview for Sexual Functioning – Self-Report, a 25-item self-report measure of sexual function for men and women [59]. It assesses five dimensions of sexuality: sexual cognition/fantasy, arousal, sexual behavior/experience, orgasm, and sexual drive/relationship. Scores can be calculated for each dimension and for global sexual functioning. The Derogatis Interview for Sexual Functioning – Self-Report demonstrates good internal consistency and reliability [59, 60].

Sexual satisfaction is measured using the Global Measure of Sexual Satisfaction Scale, which consists of five items assessing sexual experiences as good versus bad; pleasant versus unpleasant; positive versus negative; satisfying versus unsatisfying; and valuable versus worthless. Internal consistency of this scale is high (alpha = 0.90), as is test-retest reliability (*r* = 0.84) [61].

Woman and partner self-reported improvement (scale of 0 (worse) to 5 (complete cure)) and treatment satisfaction (scale of 0 (completely dissatisfied) to 10 (completely satisfied)) are measured post-treatment and at 6 months follow-up to assess the clinical significance of results.

### Data collection and management

#### Evaluation

Using the aforementioned measures, evaluations are conducted at three time points: (1) pre-treatment; (2) post-treatment (immediately following the end of treatment); and (3) 6 month follow-up (6 months following the end of treatment). Each time point includes a structured interview and standardized questionnaires. The structured interview covers demographics, gynecologic history, and includes the measures of self-reported pain during intercourse, frequency of intercourse and co-morbid pain conditions. Participants are monitored for the use of other treatments at each evaluation. Self-report questionnaire data are collected using Qualtrics Research Suite online software (Qualtrics, Provo, Utah, USA), to allow for direct entry of participants’ responses to questionnaires. Qualtrics servers are protected by high-end firewall systems, and vulnerability scans are performed regularly. Qualtrics can be used by entities that are required to comply with Health Insurance Portability and Accountability Act privacy rules.

#### Compliance

In order to ensure maximum rate of participation in all follow-up evaluations, several strategies are being implemented: (1) participants are reminded of their appointments by a research assistant; (2) participants are asked to provide several points of contact, including phone number(s), e-mail address and mailing address for both women and partners; (3) participants are given a pamphlet highlighting the importance of their continued participation; (4) participants receive a remuneration of $30.00 at each evaluation for their travel costs; and (5) follow-up appointments are scheduled at participant’s convenience (for example, on evenings and weekends). These strategies are intended to increase compliance with each phase of the protocol.

### Statistical considerations

#### Sample size, power, and statistical analysis methods

Sample size calculations were based on realistic effect sizes and average pain reductions yielded by pilot work, previous clinical trials, and observations made during previous studies focusing on different treatments for PVD [27, 38]. From the published pilot data testing CBCT, the data show effect sizes as small as d = 0.32. Therefore, using an effect size d = 0.32 (f = 0.16), *P* < 0.05, two groups, three times of measurement and a moderate correlation between repeated measures, a sample size of 124 is necessary to detect this effect with adequate power (95%) for this trial’s primary and secondary research questions. Based on previous work, a potential drop-out rate of 27% at the 6 month follow-up has been added to the sample size calculation, resulting in a total sample of 170 participants (124/0.73). Sample size calculations were conducted using GPower 3.1.3 (Heinrich Heine University Düsseldorf, Düsseldorf, Germany) [62].

#### Statistical analyses

Data storage and analysis is conducted using IBM SPSS 21.0 statistical software (Armonk, New York, USA). The suitability of variables for analysis is first examined by inspecting the univariate and multivariate normality of distributions. An estimator suitable for multivariate abnormality is chosen if necessary (robust maximum likelihood or weighted least squared estimators). Descriptive statistics of outcome variables are then compiled.

### Primary analyses

In accordance with the intent-to-treat design, all participants in their randomized group are included for the primary and secondary analyses. As some attrition is possible in this longitudinal design, missing data is handled using the full-information maximum likelihood to allow the use of all data available, even incomplete cases. To attain the main objective, which is to compare the efficacy of CBCT versus lidocaine on pain during intercourse post-treatment, a piecewise growth curve modeling approach is used [63]. In such a model, the change measured over time on the target variable is modeled within a growth curve where two stages are defined (as growth is expected to be different between pre- and post-treatment, than with the follow-up) instead of one as in a typical growth curve model. The dependent variable is then operationalized as the slope and intercept of those same variables for both stages. The model allows for testing the difference between the growth parameters in both treatment arms, and thus permits estimating the difference in change in both treatments with 95% confidence interval.

### Secondary analyses

A similar strategy is used to assess the differences between treatment groups post-treatment and at 6 months follow-up for women and partners’ sexuality (sexual function and satisfaction, frequency of intercourse), psychological adjustment (anxiety, depression, catastrophizing, self-efficacy, attributions and quality of life), relationship factors (partner responses and dyadic adjustment) and self-reported improvement and treatment satisfaction. A more conservative significance level (alpha of 0.01) is used to account for the increased number of analyses. With the addition of interaction terms, childhood trauma and co-morbid pain conditions are also planned to examine moderation of treatment response. Moreover, it is also planned to use the data from this trial for theory-testing, conducting exploratory analyses examining the extent to which changes in partner responses, catastrophizing and self-efficacy predict changes in pain and sexuality outcomes.

### Ethical aspects

This research study has been evaluated and approved by the respective ethics committees at each recruitment site. Research coordinators for each site are ensuring that the study is maintained in concordance with ethical standards of both sites. All potential participants are informed that their decision to participate or not has no impact on their medical care. Couples who choose not to participate, or who do not satisfy treatment eligibility criteria are referred for appropriate treatment if interested. Informed consent is obtained from all participants. The financial compensation that is offered to participants for their time at evaluation time-points was determined to facilitate attendance, but not to induce compliance. This trial is registered at clinicaltrials.gov NCT01935063.

## Discussion

This is the first randomized trial evaluating the efficacy of a treatment option for PVD in which the partner is included. The study of interpersonal factors in the experience of PVD has been neglected when in fact it is the most ‘interpersonal’ of pain conditions. Limitations of previously published PVD treatment research include poor participant selection, limited follow-up and a dearth of RCTs [22, 33]. Few randomized studies have evaluated behavioral and cognitive-behavioral interventions [27, 36], particularly as compared to standard forms of care. The use of a RCT design will provide a rigorous test of efficacy and high level of evidence. The two interventions being evaluated, CBCT and topical lidocaine, were developed using empirical findings and previously established treatment procedures. Both treatment protocols are standardized to facilitate uniformity in delivery to all participants, and therefore improve treatment reliability.

There are some limitations to consider with the present study design. A separation of the psychosocial and biomedical approach to treating PVD contradicts recommendations made in the literature concerning a multimodal approach to care, yet this separation is necessary to determine the efficacy of each intervention and is particularly important in the testing of the newly developed CBCT. Similarly, while a homogeneous sample of women with PVD who are sexually active may not be representative of all women and couples experiencing pain during sexual intercourse, it is the homogeneity that allows the interventions to target similar symptoms for all participants and the current sexual activity that allows for the assessment of the primary endpoint: pain experienced during sexual intercourse. Finally, this RCT does not utilize a double-blind procedure, or a control condition. Given the nature of the interventions being compared (psychosocial and biomedical), it is not possible for participants to be blinded to their assigned treatment. Comparing the CBCT to a “placebo therapy” is difficult to conceptualize and would not have been ethical as a “placebo therapy” would have required a substantial time investment from participants with potentially very limited benefits. And while wait-list control conditions were considered, it was thought to be unethical to withhold active treatment from women in pain.

This clinical trial addresses the urgent need for empirically validated treatments for PVD, the most frequent type of vulvodynia. The results will provide PVD couples with scientifically based treatment options, which may allow them to reduce their pain and improve their sexual functioning, psychological well-being and relationship. Moreover, findings from this study may be applicable to populations coping with sexual dysfunction related to health concerns.

## Trial status

Both research sites for this trial are actively recruiting participants. The trial is ongoing and has a planned duration of 3 years, with recruitment running from March 2014 to March 2017.

## Abbreviations

CBCT: cognitive-behavioral couple therapy
CBT: cognitive behavioral therapy
EASQ: Extended Attributional Style Questionnaire
IMMPACT: Initiative on Methods, Measurement, and Pain Assessment in Clinical Trials
MPI: Multidimensional Pain Inventory
MPQ: McGill Pain Questionnaire
PVD: provoked vestibulodynia
RCT: randomized clinical trial
SRI: Spouse Response Inventory.
